# Methods for detection of *Helicobacter pylori* from stool sample: current options and developments

**DOI:** 10.1007/s42770-021-00589-x

**Published:** 2021-08-15

**Authors:** Enming Qiu, Zhou Li, Shuai Han

**Affiliations:** General Surgery Center, Department of Gastrointestinal Surgery, Zhujiang Hospital, Southern Medical University, Haizhu District, No.253. Gongye Middle Avenue, Guangzhou, Guangdong, 510280 China

**Keywords:** Helicobacter pylori, Stool sample, Stool antigen test, Polymerase chain reaction, Diagnostic method

## Abstract

Accurate detection of *Helicobacter pylori* infection and determination of antibiotics have significant meaning in clinical practice. The detection methods can be categorized into two types, invasive and non-invasive, but nowadays we use the urease breath test most frequently which is non-invasive. However, many developing countries cannot meet the requirements for having specialized equipment and they lack trained personnel. Also, for the children, it is difficult to make them cooperate for the test. Methods that detect *Helicobacter pylori* from stool sample can be a promising alternative for detection used in children and mass screening. Stool antigen tests have several advantages such as rapidity, simplicity, and cheapness, though their results may be influenced by the heterogenicity of antigens, the nature of biochemical techniques, and the amount of antigen presented in the stool. PCR-based methods can specifically detect *Helicobacter pylori* infection and antibiotic resistance by targeting specific gene sequence, but they also are limited by the requirements of facilities and experts, the existence of inhibitory substance, and interference from the dead bacteria. Some novel methods also deserve our attention. Here we summarized the results of researches about methods using stool sample and we hope our work can help clinicians choose the appropriate test in clinical practice.

## Introduction

*Helicobacter pylori* (*H. pylori*) is a gram-negative pathogenic bacterium that was defined by Marshall and Warren [1], and this bacterium is responsible for several diseases, such as chronic gastritis, peptic ulcer, and gastric cancer [2]. Although most of *H. pylori*–infected patients are asymptomatic and only a small proportion of these patients will progress to peptic ulcer and gastric cancer after long-term infection [3], it still became one of the main causes of high mortality and morbidity worldwide. It is estimated that 50% of global population was infected by *H. pylori* [4, 5] which may lead to the increase of gastric cancer patients. Therefore, we need rapid, accurate, and convenient diagnostic methods to detect the *H. pylori* infection for the primary prevention of gastric cancer.

For decades, we have already developed several tools for detection of *H. pylori* and we can simply classify them as invasive methods and non-invasive methods [6]. Invasive methods include histology, culture, and rapid urease test whereas non-invasive methods include urea breath test, stool antigen test, serological test, and polymerase chain reaction (PCR). Because invasive methods cause discomfort for the patients, non-invasive methods are more commonly used in clinical practice. Detection of *H. pylori* from stool sample is non-invasive and easy to perform in epidemiological studies and diagnosis of infection in children. However, few review articles focus on these methods. Here we review the research about methods for detection of *H. pylori* from stool sample and the current progress (Table 1).

**Table 1:** Brief description for advantages and disadvantages of both methods

Stool antigen test	Polymerase chain reaction		
Advantages	Disadvantages	Advantages	Disadvantages
Easy to sampling	Difference among antigens	Detection of virulence factor	Requirements of specialized equipment and trainees
Easy to transport	Low concentration of antigen in stool	Detection of antimicrobial resistance	False positive results caused by DNA from dead bacteria
Inexpensive	Subjective interpretation	Rapid testing of large samples	False negative results caused by DNA mutations

## Stool antigen test

Stool antigen test (SAT) is a method used for direct detection of *H. pylori* antigen which exists in the feces. The diagnostic performances of different SATs are heterogeneous, and this may relate to the designs of the test like enzyme immunoassay (EIA) and immunochromatographic assay (ICA) and for the selection of antibody, such as monoclonal antibody and polyclonal antibody. Researchers have studied a lot to find out which test has the better diagnostic performance.

Some researchers assessed few of the commercial kits available recently. Halland et al. [7] assessed the performance of two novel enzyme immunoassays, the H. PYLORI QUIK CHEK™ and the H. PYLORI CHEK™ assays. They demonstrated that the sensitivities of these two detection kits were 92% and 91%, respectively; the specificity of these assays was 91% and 100%, respectively. Furthermore, they did not observe any cross-reactivity against other gut pathogen. Fang et al. [8] enrolled 347 adult subjects including 152 volunteers and 195 symptomatic patients, and they reported that the sensitivity, specificity, and accuracy of the Vstrip HpSA were 91%, 97%, and 95.7% respectively, which makes it a potential method for mass screening of *H. pylori* infection. Opekun et al. [9] evaluated the automated LIAISON® Meridian *H. pylori* SA assay, a chemiluminescent immunoassay. They combine different methods including histology, culture, and rapid urease detection test and took that as standard, and finally they compare these results with automated LIAISON® Meridian *H. pylori* SA assay results; among these two the latter indicated a great sensitivity and specificity (95.5%, 97.6%, respectively) which ultimately showed good agreement with the standards.

Several researches have demonstrated that stool antigen test could give more information to clinician and they can extend its application. Kakiuchi et al. [10] evaluated *H. pylori* antigen detection kit named Quick Chaser *H. pylori* (QCP) which targeted the flagellar protein of *H. pylori*, while other commercial kits usually targeted the catalase of *H. pylori*. They compared QCP with another stool antigen detection kit such as Testmate rapid pylori antigen (TRP) while using the rapid urease test and culture as standard. They found that the QCP had great sensitivity and specificity for detection of *H. pylori* infection and it was also sensitive to clarithromycin (CAM)–resistant isolates and CAM-susceptible isolates.

Moon et al. [11] showed that *H. pylori* antigen presented in stool was independently associated with a higher serum pepsinogen (PG) I level and a lower serum PG I/II ratio, which indicate that the patient has an active current *H. pylori* infection with increased gastric secreting ability. Yan et al. [12] showed that the SAT has the same diagnostic value in patients with distal gastrectomy as in patients without surgery.

The comparison of diagnostic performance among different stool antigen tests attracted many researchers’ interests. Zhou et al. [13] conducted a meta-analysis including 45 studies, and they concluded that SAT using ELISA monoclonal antibodies is an efficient test for the diagnosis of infection in children, yet the available polyclonal SAT tests may still be unreliable. Korkmaz et al. [14] compared the performance of 5 HpSA assay for diagnosing *H. pylori* infection in symptomatic adult patients before they accepted eradication therapy and found that Premier Platinum HpSA Plus test was the most accurate testing for the diagnosis of *H. pylori* infection, with 92.2% for sensitivity and 94.4% for specificity.

The guidelines for the management of *Helicobacter pylori* infection in Japan suggested that the SAT has great diagnostic performance, with sensitivity of 96–100% and specificity of 97–100% before eradication [15].

Conclusively, SATs have several advantages in detection of *H. pylori* infection. We can rather easily get the stool samples non-invasively which makes it possible for mass screening in a community including detection in the children. Even the stool sample can be stored at freezing temperatures whereas using commercial detection kit9 it can be stored at ambient temperature for a long time, which makes it easy to transport. Since SATs do not require expensive chemical agents and special equipment, its price is lower than other non-invasive methods like urease breath test. Among the biochemical design of SATs, ICA-based tests are potential point-of-care detection kits since it is easy to perform and rapid for detection and hence it is a good alternative method for frontline medical staff in public health centers without laboratories [16].

Despite the advantages as mentioned above, SATs also have several disadvantages. Since the principle of SAT is an antigen–antibody reaction, differences in antigens used for SAT in different geographic regions may cause heterogeneity of results [17]. We should be conscious that the negative result of SAT may not indicate the absence of *H. pylori* infection because the low colonization of bacteria in stomach leads to low concentration of *H. pylori* antigen in the sample [18]. Regarding ICA-based methods, subjective interpretation of the results, especially for the tracing line, makes it difficult to diagnose accurately [13, 19].

We should also be cautious that in some special situations, the sensitivity of SAT may decrease, such as those for patients with gastrointestinal bleeding or for patients undergoing bismuth-based therapy [14].

## Polymerase chain reaction

Polymerase chain reaction (PCR) is a reliable technology which is widely used in biology and medical research. Use of PCR for detection of *H. pylori* infection attracts many researchers’ attention. Nowadays, there are several commercial detection kits available based on PCR. The common methods used for detection are real-time PCR, nested-PCR, and droplet digital PCR (ddPCR).

Some researchers have evaluated the diagnostic performance of commercial detection kits. Pichon et al. [20] assessed the performance of the Amplidiag *H. pylori* ClariR assay in detecting *H. pylori* infection and clarithromycin resistance in a large cohort study, and they demonstrated the good diagnostic performance of the assay in both of the aspects mentioned above. Redondo et al. [21] reported the diagnostic performance of commercial kits named LightMix® RT-PCR assay. This assay analyzed the melting curve to achieve simultaneous detection of *H. pylori* infection and clarithromycin resistance.

One of the important areas where PCR-based assay can be applied is the detection of antibiotic resistance and virulence factor, since antibiotic therapy is the foundation of the triple therapy for *H. pylori* eradication and the virulence factors are important for our clinicians to evaluate the infection. Rolon et al. [22] reported that apart from the high sensitivity and specificity of PCR-based assay, it may also be able to predict the prognosis of eradication treatment. The clarithromycin triple therapy was more likely successful when there is no prediction of resistance. Sun et al. [23] established a testing based on ddPCR to simultaneously detect *H. pylori* clarithromycin-resistant (mutant) and clarithromycin -susceptible (wild-type) 23S rRNA gene alleles in stool sample. Beckman et al. [24] also demonstrated that PCR-based assay can detect the presence of *H. pylori* DNA and mutations associated with resistance against clarithromycin at the same time. They also proposed two approaches to determine the existence of clarithromycin resistance. Talarico et al. [25] applied ddPCR-based *H. pylori* detection to disclose the relationship between significant variation in bacterial load among individuals and presence of the cagA virulence gene.

Another important application for PCR-based methods is to determine the prevalence of *H. pylori* infection and antimicrobial resistance in children. Beer-Davidson et al. [26] enrolled 188 samples from schoolchildren aged 6–9 years and 272 samples from healthy infants to develop and validate a multiplex real-time polymerase chain reaction (q-PCR) assay. They also evaluated the prevalence of clarithromycin resistance and cagA gene in *H. pylori*–positive samples and confirmed them with gene sequencing. Sicinschi et al. [27] used PCR-based methods to genotype alleles associated with virulence factors from stool sample of healthy Colombian children living in an area with high prevalence of gastric cancer. They revealed the high prevalence of disease-associated genotypes, such as cagA, hopQ, and vacA, and their results may prove that PCR-based methods can be used to screen for relevant genotypes in stool samples from a population. Scaletsky et al. [28] validate a novel bi-probe real-time assay in stool specimens from 217 dyspeptic children for *H. pylori* clarithromycin susceptibility testing, and Mishra et al. [29] also used nested-PCR to determine the prevalence of *H. pylori* in asymptomatic children.

Technology improvement is always the target for us in order to get more accurate diagnostic performance. Leonardi et al. [30] wondered whether the bead-beating step prior to DNA extraction can enhance the amount of DNA extracted, yet the results did not seem to support their hypothesis.

PCR-based *H. pylori* detections have several advantages. They can detect *H. pylori* infection and antimicrobial resistance simultaneously in short time. However, they have not been accepted for the routine testing like UBT because they have several disadvantages. First of all, PCR-based methods require specialized equipment and trained personnel which may not be easy for lower-income countries. Secondly, the concerns about the false-negative results and false-positive results still exist, since the PCR targets the DNA of active bacteria but DNA from dead bacteria may cause a false-positive result, especially after the eradication treatment. As we know, *H. pylori* can transfer to coccoid form when it is difficult for them to survive in the environment [31] and this characteristic may obstruct the accurate detection of PCR-based methods [32]. The increasing presence of coccoid form and existence of mutation may lead to the false-negative result. The last but not the least is that some substances in the stool sample such as hemoglobin and its degradation products, polysaccharide complexes, heavy metals, and proteins, may inhibit the PCR-based testing. A research reported that the THD Fecal Test can eliminate PCR inhibiting substances such as polysaccharide complexes [33].

## Novel methods for detection from stool sample

Apart from stool antigen test and PCR, there are some novel techniques developed recently. Ali et al. [34] reported a colorimetric paper device which can detect *H. pylori* sensitively and specifically. Their device was designed on the basis that the DNAzymes can specifically cleave RNA and can be activated by the crude extracellular mixture of *H. pylori*. What makes this device more meaningful is that it can be stored at ambient temperature for at least 130 days and still remain fully functional. Chen et al. [35] provided a new non-invasive detection method based on the combination of immunomagnetic beads and antigen–antibody reaction. The immunomagnetic beads conjugated with monoclonal antibodies sensitively recognized and captured the *H. pylori*, and the complex can be coupled with a polyclonal antibody-conjugating quantum dot probe. After that, fluorescence spectrometer was used to achieve ultrasensitive detection. Jain et al. [36] utilized a clustered metallic nanoparticle along with carbon nanomaterial and conducted polymer-based ultrasensitive immunosensor for *H. pylori* detection. Their device provided high loading of CagA antibody and resulted into a great diagnostic performance. All these explorations broaden our horizons and provide more possibility for accurate diagnosis of *H. pylori* infection.

## Discussion

*H. pylori* infection is the cause of many gastroduodenal diseases and over half of the population worldwide are infected with it. Ding et al. [37] conducted a prospective, cross-sectional, population-based study involving 3491 children (0–18 years) in total. They found that the overall infection rate was 6.8% with no significant differences between genders. Infection rates between regions were significantly different (*P* < 0.05) and increased with age. Because of the great prevalence of *H. pylori* infection, increasing infection rate with age, and the potential pathogenicity, it is important to detect *H. pylori* and antibiotic resistance accurately both in adult and children whether they are symptomatic or not.

There are several methods for detection of *H. pylori* infection and nowadays we usually utilize UBT as the first choice of detection. However, it has several disadvantages, such as the need for expensive equipment and reagents and well-trained experts to interpret the results, which limits its application. Also, UBT does not perform well in children because it may be difficult for young children and infants to cooperate. Therefore, detection based on stool sample would be a potential and promising alternative for detection of *H. pylori* infection and the mass screening.

Detections for *H. pylori* from stool sample have several points worthy of our attention. For SAT, firstly, we should be cautious about the shape of stool and the interval between sample collection and detection. Watery feces may provide false‐negative results because of antigen dilution. Assessments using excreted feces become impossible 24–48 h after collection, but this may be solved by using a robust sampling kit. Secondly, we should better conduct local validation of antigen used in SAT if conditions permit [15]. Thirdly, we should be careful about patients with severe atrophic gastritis and intestinal metaplasia. Because of the decreased colonization of *H. pylori*, it may lead to a false-negative result [38]. Although most studies have shown that the effect of PPI on SAT is less effective on UBT [39, 40], we still recommend that clinicians carefully evaluate the impact of ongoing treatments that include PPI, antibiotics, and bismuth. In addition, the interval between drug withdrawal and testing should also attract our attention.

Regarding of PCR-based methods, we should be worried about the quality and amount of DNA extracted, the design of target sequence, and the selection of amplification protocol. A progress which can stabilize the *H. pylori* DNA and enhance the amount of the extract may help a lot. It is suggested that nested-PCR is more sensitive than the regular PCR because it involves two rounds of amplification, which makes it able to amplify the target sequence in a lower concentration [41]. What’s more, combination of several target genes for detection, such as ureA, glmM, and vacA, may help to improve the diagnostic performance by reducing the possibility of missed detection [42].

For all the testing used stool specimens, we should be aware of external contamination because it can interfere with the normal results. Another point worthy of our attention is that gastrointestinal tracts in children and those of adults are different, such as composition of gut flora and passage time of stool [43].

## Conclusion

Detection of *H. pylori* infection and antibiotic resistance is an important issue for prevention of several gastroduodenal diseases like gastric cancer. Detections from stool sample have several advantages such as simplicity of sample collection yet they also have many limitations. In this review, we summarized what the researchers have reported, the new progress in methodology, and some aspects we should pay attention to when we utilize these methods. We hope this review can help clinicians choose the suitable technique for detection in their clinical practice.

## Data Availability

Not applicable.
